# A Genetically Engineered Biomimetic Nanodecoy for the Treatment of Liver Fibrosis

**DOI:** 10.1002/advs.202405026

**Published:** 2024-08-29

**Authors:** Yang Du, Hao Ding, Yining Chen, Bingqiang Gao, Zhengwei Mao, Weilin Wang, Yuan Ding

**Affiliations:** ^1^ Department of Hepatobiliary and Pancreatic Surgery The Second Affiliated Hospital Zhejiang University School of Medicine Hangzhou 310009 China; ^2^ Key Laboratory of Precision Diagnosis and Treatment for Hepatobiliary and Pancreatic Tumor of Zhejiang Province Hangzhou 310009 China; ^3^ Research Center of Diagnosis and Treatment Technology for Hepatocellular Carcinoma of Zhejiang Province Hangzhou 310009 China; ^4^ National Innovation Center for Fundamental Research on Cancer Medicine Hangzhou 310009 China; ^5^ Cancer Center Zhejiang University Hangzhou 310058 China; ^6^ ZJU‐Pujian Research and Development Center of Medical Artificial Intelligence for Hepatobiliary and Pancreatic Disease Hangzhou 310058 China; ^7^ MOE Key Laboratory of Macromolecular Synthesis and Functionalization Department of Polymer Science and Engineering Zhejiang University Hangzhou 310027 China; ^8^ State Key Laboratory of Transvascular Implantation Devices Hangzhou 310009 China

**Keywords:** biomimetic nanodecoy, cellular nanovesicle, drug delivery, genetic engineering, liver fibrosis

## Abstract

Liver fibrosis, arising from factors such as viral infections or metabolic disorders, represents an ongoing global health challenge and is a major risk factor for hepatocellular carcinoma. Unfortunately, there are no clinically approved drugs available for its treatment. Recent studies have illuminated the pivotal role of macrophage recruitment in the pathogenesis of liver fibrosis, presenting a potential therapeutic target. Therefore, it holds great promise to develop novel anti‐fibrotic therapies capable of inhibiting this process. Herein, a drug‐loaded biomimetic nanodecoy (CNV‐C) is developed by harnessing genetically engineered cellular vesicles for the treatment of liver fibrosis. CNV‐C is equipped with a C‐C motif chemokine receptor 2 (CCR2)‐overexpressed surface, enabling it to selectively neutralize elevated levels of C‐C motif chemokine ligand 2 (CCL2), thereby reducing macrophage infiltration and the subsequent production of the fibrogenic cytokine transforming growth factor β (TGF‐β). Moreover, curcumin, an anti‐fibrotic agent, is loaded into CNV‐C and delivered to the liver, facilitating its efficacy in suppressing the activation of hepatic stellate cells by blocking the downstream TGF‐β/Smad signaling. This combinational therapy ultimately culminates in the alleviation of liver fibrosis in a mouse model induced by carbon tetrachloride. Collectively, the findings provide groundbreaking proof‐of‐concept for employing genetically modified nanodecoys to manage liver fibrosis, which may usher in a new era of anti‐fibrotic treatments.

## Graphical abstract

This study introduces a novel curcumin‐loaded nanodecoy (CNV‐C) for treating liver fibrosis. By utilizing C‐C motif chemokine receptor 2 (CCR2)‐overexpressing cellular nanovesicles to neutralize C‐C motif chemokine ligand 2 (CCL2) and deliver curcumin, CNV‐C effectively reduces macrophage infiltration and hepatic stellate cell activation. This two‐pronged strategy highlights the potential of genetically modified nanodecoys as an effective treatment for liver fibrosis.

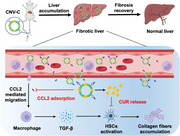

## 1 Introduction

The chronic liver disease (CLD) has gradually become a major health problem in the world, and it is one of the most common causes of death, resulting in ≈2 million deaths per year.^[^ ^1^ ^]^ Liver fibrosis is an important pathological change in liver injury caused by various CLDs, including hepatitis B and C, alcoholic steatohepatitis, nonalcoholic steatohepatitis, biliary obstruction, and several other etiologies.^[^ ^2^ ^]^ If the process of liver fibrosis cannot be effectively controlled, it may eventually develop into liver cirrhosis and even liver failure and hepatocellular carcinoma.^[^ ^3^ ^]^ At present, there exists no approved drug therapy for liver fibrosis, underscoring the urgent need for the development of innovative and efficacious pharmacological interventions.

The process of liver fibrosis is accompanied by decreased degradation and increased deposition of extracellular matrix (ECM) proteins, gradually forming fibrous scars to replace damaged liver tissue.^[^ ^2^ ^]^ The hepatic stellate cells (HSCs) are the main precursors of ECM‐producing myofibroblasts, which contribute 82–96% of myofibroblasts in various types of CLDs, and are the key to the occurrence and development of liver fibrosis.^[^ ^4^ ^]^ Recently, the inhibition of HSC activation has garnered significant attention as an effective therapeutic strategy for mitigating liver fibrosis.^[^ ^5^ ^]^ However, the activation of HSCs is not an isolated process but also depends on its interaction with other cells. Macrophages are one of the important cells that participate in the progression of liver fibrosis.^[^ ^6^ ^]^ Peripheral monocytes could be recruited in the liver by chemokines, notably C‐C motif chemokine ligand 2 (CCL2), and mature into pro‐fibrogenic macrophages, which release fibrogenic cytokines such as transforming growth factor β (TGF‐β) to promote the activation of HSCs.^[^ ^7^ ^]^ The up‐regulation of CCL2 has been observed in various CLDs, and the inhibition of CCL2/C‐C motif chemokine receptor 2 (CCR2) signaling reduces monocyte‐derived macrophage infiltration and attenuates liver fibrosis.^[^ ^8^ ^]^ Blocking the CCL2/CCR2 axis can weaken the bridge between liver inflammation and HSC activation, which provides new possibilities for the treatment of liver fibrosis.^[^ ^9^ ^]^ Currently, several CCR2 inhibitors have demonstrated anti‐fibrotic effects in animal models of live fibrosis.^[^ ^10^ ^]^ However, the clinical trials of cenicriviroc, the only CCR2 inhibitor undergoing testing, failed to achieve the primary outcomes and reported relatively low numbers of patients benefiting from the treatment.^[^ ^11^ ^]^ Therefore, achieving precise and effective blocking of the CCL2/CCR2 signaling pathway in liver fibrosis remains a significant challenge.

In recent years, cell membrane‐based nanovesicles have emerged as promising therapeutic agents for addressing inflammation‐related diseases by regulating inflammatory mediators within the microenvironment.^[^ ^12^ ^]^ These nanovesicles offer unique advantages as they inherit surface characteristics from their source cells, enabling them to simulate receptor‐ligand interactions without the need for complex chemical treatments or traditional synthetic modifications.^[^ ^13^ ^]^ They can function as nanodecoys, neutralizing pathological molecules involved in disease progression.^[^ ^14^ ^]^ Notably, the cell membrane surface can be genetically engineered to incorporate specific receptors for the capture of pathogenic factors.^[^ ^15^ ^]^ Consequently, the fabrication of nanovesicles overexpressing CCR2 holds the potential to efficiently counteract the pathogenic effects of excessive CCL2 in fibrotic livers. Moreover, nanovesicles derived from cell membranes possess a phospholipid bilayer structure that allows for the incorporation of hydrophobic drugs into the bilayer, thereby enhancing their solubility and bioavailability.^[^ ^16^ ^]^ Considering the intricate pathogenesis of liver fibrosis, there is a high demand for combination therapy with other anti‐fibrotic drugs that can simultaneously target different pathways and achieve synergistic therapeutic effects.

**Figure 1:**
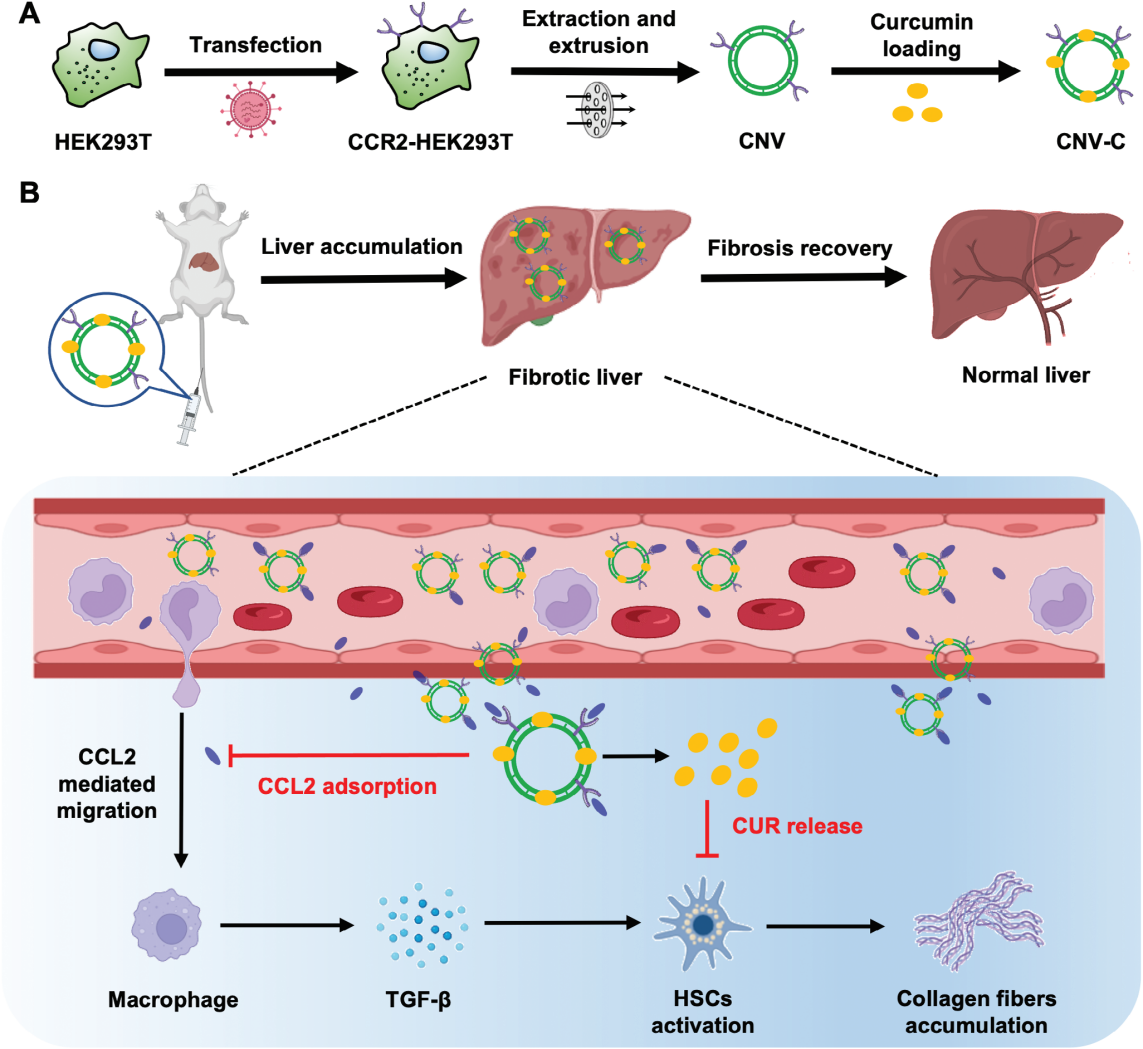
Schematic illustration of the CNV‐C preparation process and the proposed strategy for treating liver fibrosis. A) HEK293T cells are transfected with CCR2‐mScarlet lentivirus to construct CCR2‐overexpressed cells. Subsequently, CNV is prepared by extracting and extruding the membranes of these CCR2‐HEK293T cells. Finally, curcumin is loaded into the lipid bilayer of CNV to form CNV‐C. B) After tail vein injection, CNV‐C can accumulate in the fibrotic liver, where it serves as a nanodecoy, efficiently adsorbing CCL2 to reduce macrophage infiltration and diminish TGF‐β production. Meanwhile, curcumin is released from CNV‐C to amplify its effectiveness in inhibiting HSC activation, thereby leading to the alleviation of liver fibrosis.

In this study, a drug‐loaded and genetically engineered biomimetic nanodecoy (CNV‐C) for the management of liver fibrosis is developed. Specifically, CCR2‐overexpressing nanovesicles (CNV) are prepared by extracting cell membranes from human embryonic kidney (HEK293T) cells transfected with CCR2. Curcumin (CUR), a natural polyphenolic compound that exhibits inhibitory effects on HSC activation,^[^ ^17^ ^]^ is further loaded into CNV to obtain CNV‐C (**Figure** **1**). After intravenous injection, CNC‐C shows remarkable accumulation and prolonged retention in the liver. It can absorb the excessive CCL2 through surface‐expressed CCR2 to reduce the infiltration of monocyte‐derived macrophages, resulting in the decreased production of fibrogenic cytokine TGF‐β. Furthermore, the sustained release of CUR from CNV‐C induces the quiescence of HSCs by directly blocking the downstream TGF‐β/Smad signaling. Consequently, CNV‐C significantly improves liver morphology and structure in tetrachloromethane‐induced liver fibrosis mice. This study introduces a feasible strategy for treating liver fibrosis using biomimetic nanodecoys, representing a promising avenue for future clinical translation.

## 2 Results and Discussion

### 2.1 Preparation and Characterization of CNV‐C

**Figure 2:**
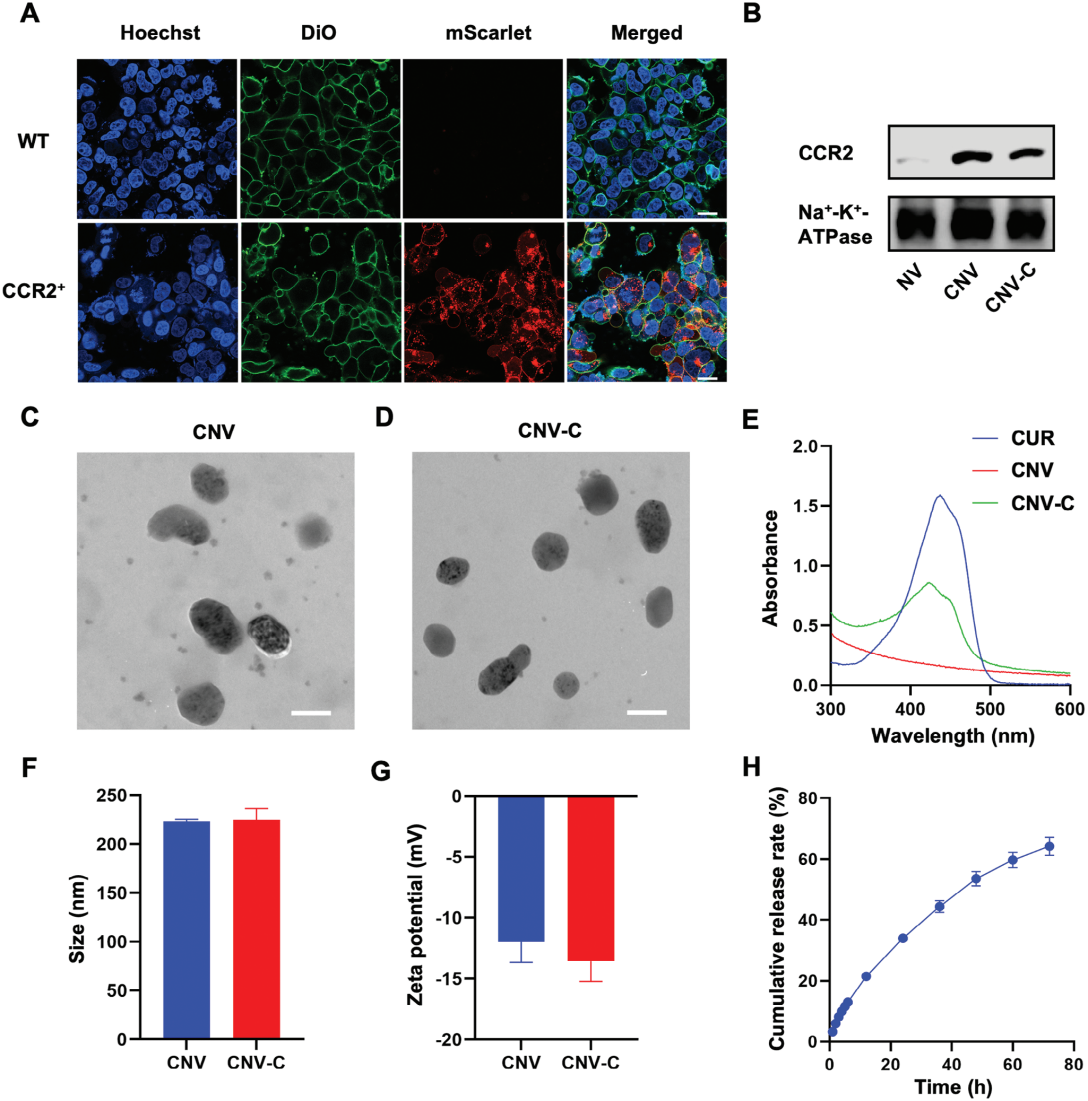
Preparation and characterization of CNV‐C. A) CLSM images of wild‐type HEK293T cells and CCR2‐HEK293T cells. DiO‐stained cell membrane (green), CCR2‐mScarlet (red), and Hoechst‐labeled nucleus (blue). Scale bar = 20 µm. B) Western blot analysis of NV, CNV, and CNV‐C. C) TEM images of CNV. Scale bar = 200 nm. D) TEM images of CNV‐C. Scale bar = 200 nm. E) UV–Vis absorption spectra of CUR in DMSO, and CNV and CNV‐C in PBS. F) Hydrodynamic size of CNV and CNV‐C in PBS (n = 3). G) Zeta potential of CNV and CNV‐C in PBS (n = 3). H) Cumulative release rate of curcumin from CNV‐C (n = 3).

To prepare CNV, a CCR2‐overexpressing stably transfected cell line (CCR2‐HEK293T) was first constructed by transfecting wild‐type HEK293T cells with CCR2‐mScarlet lentivirus. HEK293T cells have been used to prepare genetically engineered cellular nanovesicles for many biomedical applications due to their rapid mitotic cycle and remarkable transfection capabilities, which yield abundant proteins of interest.^[^ ^18^ ^]^ Therefore, in this work, HEK 293T cells were selected for the preparation of CNV‐C. As shown in **Figure** **2**A, the successful transfection was validated by the presence of colocalized fluorescence signals from mScarlet reporter proteins (red) and DiO‐stained cell membranes (green) using confocal laser scanning microscopy (CLSM). We noted that the fluorescence of CCR2‐mScarlet could be observed both on the cell membrane and in the cytoplasm. This may be attributed to the transport of CCR2‐mScarlet from the cytoplasm to the cell membrane during protein synthesis.^[^ ^19^ ^]^ Then, the cell membranes of CCR2‐HEK293T cells were separated by freeze‐thaw treatment and centrifugation. The membranes were collected and shaped through porous polycarbonate membranes with pore sizes of 400 and 200 nm by a liposome extruder to obtain CNV. Western blot analysis showed that CCR2 was expressed on CNV compared with nanovesicles prepared from wild‐type HEK293T cells (denoted as NV) (Figure 2B). The morphology of CNV was confirmed by transmission electron microscopy (TEM), showing a vesicle‐shaped structure with a hydrodynamic diameter of 223.0 ± 2.2 nm (Figure 2C,F). Finally, leveraging the lipophilic nature of CUR, it could be loaded into the lipid bilayer of CNV to form a yellow suspension (CNV‐C) (Figure S1A, Supporting Information). The UV–Vis spectrum of the prepared CNV‐C showed a characteristic absorption peak at 436 nm (Figure 2E), indicating successful CUR loading. The concentration of CUR could be further quantified according to its absorbance, which was linearly related to its concentration (Figure S1B, Supporting Information). The optimal drug loading capacity of CUR was found to be 45.3 ± 0.7%, and the corresponding encapsulation efficiency was 82.8 ± 2.2% (Figure S1C,D, Supporting Information). Moreover, CNV‐C maintained a complete vesicle structure after loading with CUR (Figure 2D). The hydrodynamic diameter and zeta potential of CNV‐C were measured to be 224.9 ± 11.6 nm and −13.6 ± 1.7 mV, respectively (Figure 2F,G), showing negligible changes after CUR loading. The expression of CCR2 on CNV‐C was also confirmed by Western blotting (Figure 2B). Notably, CNV‐C exhibited the capability for controlled drug release, with 64.1 ± 3.0% of CUR released within 72 h (Figure 2H), suggesting the potential of CNV‐C as a sustained‐release vehicle for CUR. In addition, we observed no significant changes in the particle size and zeta potential of CNV‐C during four days of storage in both PBS and DMEM supplemented with 10% fetal bovine serum (Figure S2, Supporting Information), indicating good colloidal stability.

### 2.2 CNV‐C Serves as CCL2 Nanodecoy to Inhibit Macrophage Infiltration In Vitro

**Figure 3:**
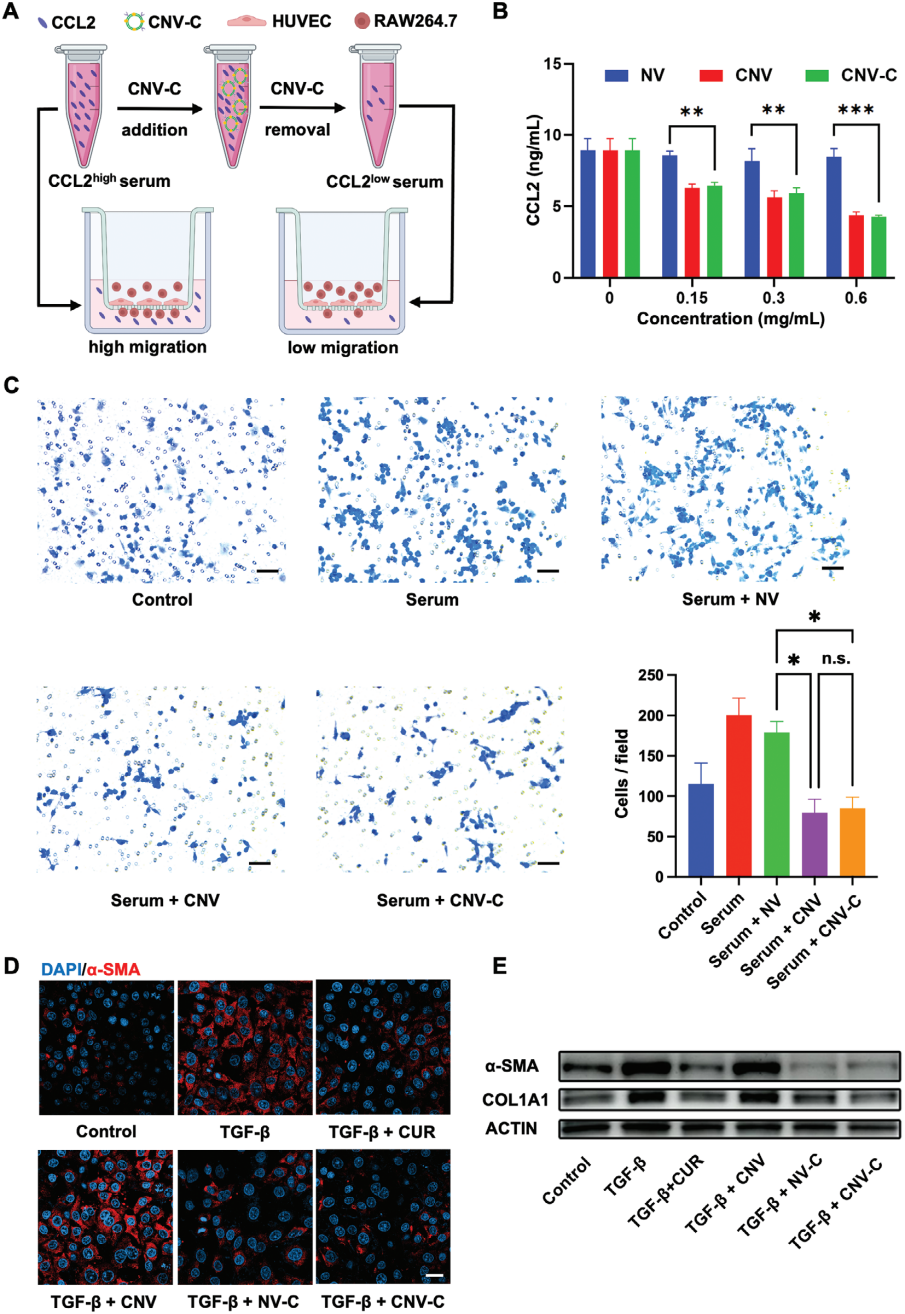
Inhibitory effects of CNV‐C on macrophage infiltration and HSC activation in vitro. A) Schematic illustration of CCL2 adsorption experiment and transwell migration assay. The CCL2^high^ serum is collected 8 h after APAP modeling in mice. The CCL2^Low^ serum is obtained by incubating CCL2^high^ serum with CNV‐C, which is subsequently removed by centrifugation before the transwell assay. B) The adsorption capacity of NV, CNV, and CNV‐C to CCL2 are examined by ELISA. The ordinate represents the concentrations of remaining CCL2 after incubation with NV, CNV, and CNV‐C (n = 3). C) Crystal violet staining of migrated RAW264.7 cells and the corresponding quantitative analysis (n = 3). Scale bar = 20 µm. D) Representative CLSM images of α‐SMA expression in LX‐2 cells after different treatments. α‐SMA (red) and DAPI‐labeled nucleus (blue). Scale bar = 20 µm. E) Western blot analysis of α‐SMA and COL1A1 expression in LX‐2 cells after different treatments. ^*^ *p* < 0.05; ^**^ *p* < 0.01; ^***^ *p* < 0.001; n.s., not significant (*p* > 0.05).

To evaluate the CCL2 adsorption capacity of CNV‐C, serum samples from a mouse model of acetaminophen (APAP)‐induced acute liver injury were acquired. It has been reported that serum CCL2 levels significantly increase in this model.^[^ ^20^ ^]^ We also observed an enhancement of serum CCL2 levels 8 h after APAP administration (Figure S3, Supporting Information). Therefore, we believe that collecting serum samples from APAP‐treated mice is an effective method to obtain serum with high CCL2 levels. The serum samples were mixed with NV, CNV, or CNV‐C, and the supernatants were collected for testing after centrifugation (**Figure** **3**A). The level of CCL2 in both the CNV group and CNV‐C group showed a concentration‐dependent reduction, with CCL2 level decreasing from 8.9 ± 0.8 to 4.4 ± 0.2 and 4.3 ± 0.1 ng mL^−1^, respectively (Figure 3B). However, the NV group, lacking CCR2 expression, displayed no significant adsorption. These results indicate that CNV‐C exhibits a CCR2‐dependent adsorption effect on CCL2. The infiltration of monocytes into the liver after injury is tightly regulated by the CCL2/CCR2 axis and contributes to hepatic inflammation and fibrogenesis.^[^ ^21^ ^]^ To verify the effect of CNV‐C on macrophage migration in vitro, we cultured RAW264.7 cells in serum pre‐treated with CNV‐C and conducted assessments using transwell assays. A layer of human umbilical vein endothelial cells (HUVECs) was seeded in the upper chamber of the transwell system to simulate the barrier effect of liver sinusoidal endothelial cells (Figure 3A).^[^ ^22^ ^]^ When cultured with CCL2‐enriched serum, the migration of RAW264.7 cells was enhanced. Notably, this chemotactic migration was significantly inhibited in the CNV group and CNV‐C group, while the NV group showed no impact on macrophage migration (Figure 3C). These findings suggest the potential of CNV‐C as a nanodecoy for effectively impeding CCL2‐mediated macrophage infiltration.

### 2.3 CNV‐C Attenuates HSC Activation In Vitro

HSCs play a key role in mediating liver fibrosis and can be activated by TGF‐β, which is released from recruited macrophages.^[^ ^23^ ^]^ To investigate the inhibitory potential of CNV‐C on TGF‐β‐induced activation of HSCs, human hepatic stellate cells (LX‐2) were exposed to TGF‐β (10 ng mL^−1^) and co‐incubated with CUR, NV‐C (curcumin‐loaded nanovesicles without CCR2 expression), CNV or CNV‐C for 24 h (30 µm for CUR). Immunofluorescence staining revealed a noteworthy reduction in the fluorescence intensity of α‐SMA, a hallmark of HSC activation, in the CUR group, NV‐C group, and CNV‐C group, indicating the inhibitory effect of CUR (Figure 3D). The CNV group showed comparable fluorescence intensity to the TGF‐β group, suggesting that CNV did not exhibit a direct inhibitory effect on HSC activation. Moreover, Western blotting also confirmed diminished expression levels of α‐SMA and COL1A1 after CNV‐C treatment (Figure 3E). Additionally, we observed that the phosphorylation of Smad3 induced by TGF‐β was inhibited in CNV‐C‐treated cells (Figure S4, Supporting Information), indicating the downregulation of TGF‐β/Smad signaling by CUR. These results collectively demonstrate that CNV‐C holds the potential as an anti‐fibrotic agent by releasing CUR for HSC inactivation.

### 2.4 CNV‐C Exhibits Improved Liver Accumulation and Satisfactory Biosafety In Vivo

**Figure 4:**
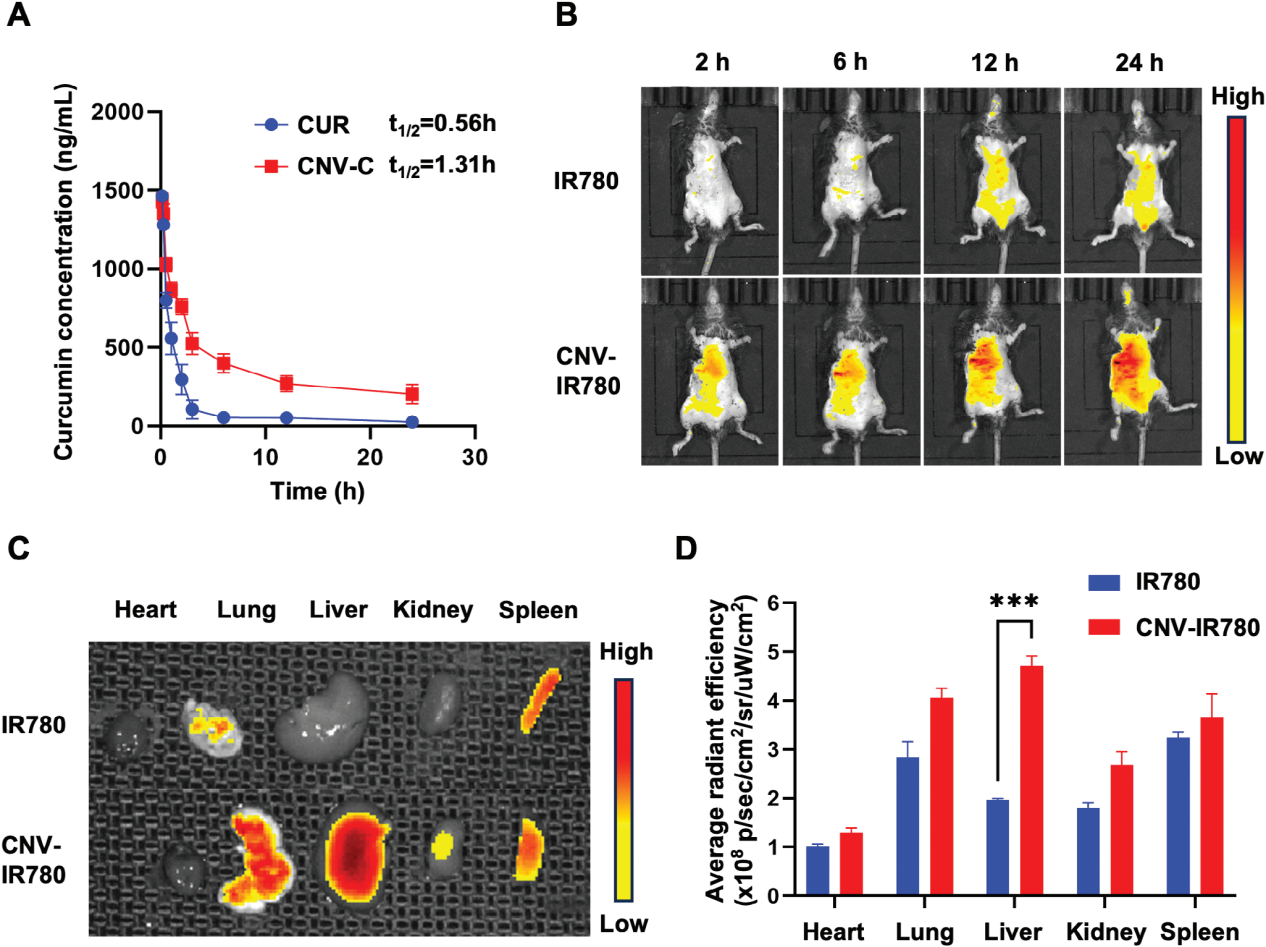
Pharmacokinetics and biodistribution of CNV‐C in vivo. A) Plasma pharmacokinetics of CUR and CNV‐C after intravenous administration in mice (n = 3). B) In vivo fluorescence imaging of mice after intravenous injection with IR780 or IR780‐labeled CNV at indicated time points. C) Representative ex vivo fluorescence imaging of major organs (heart, lung, liver, kidney, and spleen) collected from mice in different groups at 24 h post‐injection. D) Quantitative analysis of the fluorescence intensity in major organs (n = 3). ^***^ *p* < 0.001.

Ensuring long circulation and effective accumulation at lesion sites are critical for drug molecules to exert their maximum efficacy.^[^ ^24^ ^]^ Therefore, the pharmacokinetics and biodistribution of CNV‐C were further investigated in C57BL/6 mice. We observed a 2.3‐fold increase in the blood half‐life of CNV‐C compared to that of free CUR (**Figure** **4**A), indicating prolonged circulation mediated by cellular nanovesicles. To visualize the biodistribution of CNV‐C, IR780‐loaded CNV (CNV‐IR780) was intravenously injected into C57BL/6 mice for in vivo fluorescence imaging. IR780 was chosen because it is a near‐infrared dye with similar hydrophobicity to CUR. The in vivo fluorescence imaging clearly showed that CNV‐IR780 tended to accumulate in the liver and lung compared to free IR780 (Figure 4B,C). This biodistribution profile may be attributed to the nano‐size effect, which has been reported in other cell‐derived nanovesicles, such as extracellular vesicles.^[^ ^25^ ^]^ Furthermore, to better quantify the accumulation of IR780 in major organs, organs were harvested for ex vivo imaging at 24 h after injection. We found that the accumulation of CNV‐IR780 in the liver was significantly higher than that of free IR780 (Figure 4C,D). This improvement in the delivery of hydrophobic drugs mediated by CNV may benefit the therapeutic response of CUR. Moreover, the cellular uptake behavior of CNV‐C in the liver was investigated through immunofluorescent staining of liver slices (Figure S5B, Supporting Information). We found that the fluorescence of CNV‐C colocalizes with markers of hepatocytes (ALB) and macrophages (F4/80), but not HSCs (α‐SMA), indicating that CNV‐C is mainly taken up by hepatocytes and macrophages. We further investigated the elimination of CNV‐C by extending the observation period of mice post‐injection using in vivo fluorescence imaging. CNV‐C was retained in the liver for up to 72 h, allowing it to exert its therapeutic effect. We found that the fluorescence of CNV‐C in the liver gradually decreased over time (Figure S5A, Supporting Information), indicating its degradation. The immunofluorescence staining of liver sections at 72 h demonstrated colocalized fluorescence between CNV‐C and macrophages (Figure S5B, Supporting Information), suggesting that CNV‐C was cleared by the mononuclear phagocyte system in the liver. A similar clearance mechanism was observed in other cell‐derived nanovesicles.^[^ ^12c^ ^]^ In addition, both the short‐term and long‐term biosafety of CNV‐C was evaluated in C57BL/6 mice. After 24 h or three weeks of administration with CNV‐C (8 mg kg^−1^ CUR twice per week), blood samples and major organs were harvested for biochemical and histopathological analyses. Hematology‐related indicators showed no noticeable changes after both single and repeated administration (Figures S6A and S7A, Supporting Information). Hematoxylin and eosin (H&E) staining results indicated the absence of abnormal changes or pathological damage in all examined tissues (Figures S6B and S7B, Supporting Information). Collectively, the nano‐size effect of CNV‐C endows it with liver‐targeted accumulation, which may reduce the risk of side effects caused by the nonspecific distribution of current small‐molecule CCR2 inhibitors. Additionally, the drug loading capacity makes CNC‐C an integrated platform for a synergistic anti‐fibrotic effect.

### 2.5 CNV‐C Alleviates Liver Fibrosis In Vivo

**Figure 5:**
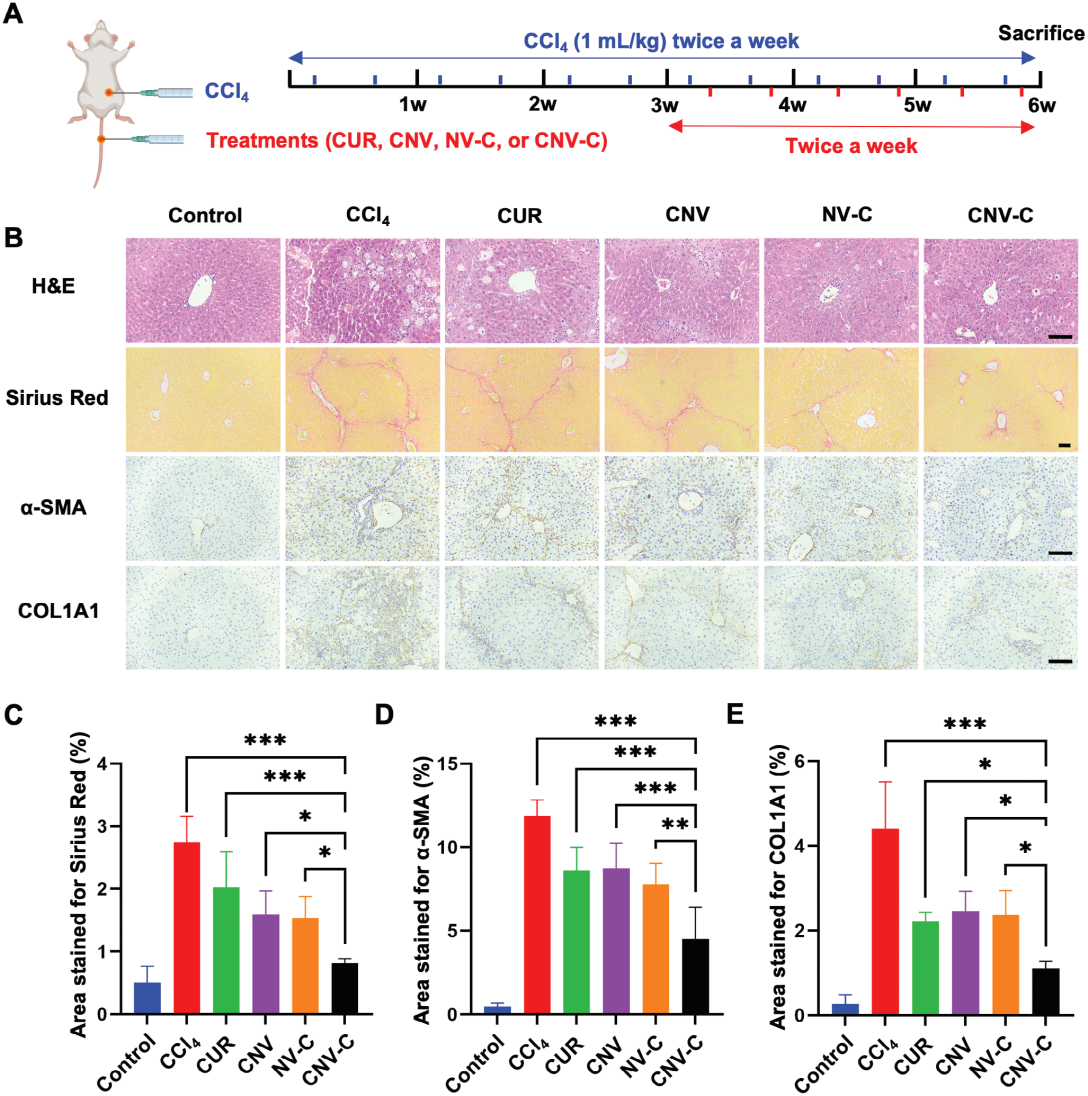
Anti‐fibrotic effects of CNV‐C in vivo. A) Treatment schedule for liver fibrosis mice induced by CCl_4_. The mice received injections of CCl_4_, CUR, CNV, NV‐C, and CNV‐C at the indicated time points and were sacrificed at the end of the sixth week. B) Representative images of H&E, Sirius Red, α‐SMA, and COL1A1staining in mice liver from indicated groups. Scale bars = 100 µm. C) Quantification of Sirius Red positive area in different groups (n = 5). D) Quantification of α‐SMA positive area in different groups (n = 5). E) Quantification of COL1A1 positive area (n = 5). ^*^ *p* < 0.05; ^**^ *p* < 0.01; ^***^ *p* < 0.001.

The therapeutic efficacy of CNV‐C in treating liver fibrosis was further explored using a mouse model induced by carbon tetrachloride (CCl_4_). C57BL/6 mice were intraperitoneally injected with 1 mL kg^−1^ of CCl_4_ twice a week for six weeks to induce liver fibrosis. After the initial three weeks of CCl_4_ administration, the mice received additional intravenous injections of the indicated formulations (8 mg kg^−1^ for CUR) twice a week for three weeks (**Figure** **5**A). Histopathological analyses using H&E staining and Sirius Red staining were employed to assess the degree of liver fibrosis in different groups. In the CCl_4_ group, extensive liver cell degeneration and necrosis were observed, accompanied by inflammatory cell infiltration and disruption of hepatic cords. Moreover, a substantial deposition of collagen fibers occurred in the portal area, leading to the formation of fibrous septa and pseudolobules. Conversely, the CNV‐C group displayed a relatively intact liver structure with reduced inflammatory cell infiltration and collagen fiber proliferation. However, only moderate relief from fibrosis was observed in the CUR group, CNV group, and NV‐C group (Figure 5B). Quantitative analysis of Sirius Red staining also showed a significantly lower percentage of positive staining area in the CNV‐C group compared to the other groups (Figure 5C), which indicates the improved anti‐fibrotic efficacy achieved by CNV‐C. Furthermore, immunohistochemical staining was applied to estimate the levels of α‐SMA and COL1A1 expression in mouse livers to detect HSC activation. CCl_4_‐induced mouse livers exhibited increased α‐SMA and COL1A1 expressions in both the portal area and liver parenchyma, which were significantly attenuated by CNV‐C treatment (Figure 5B). These findings were further confirmed by the quantification of α‐SMA and COL1A1 positive areas (Figure 5D,E), as well as Western blotting analysis (Figure S8A, Supporting Information). In addition, we found a significant down‐regulation in the gene expression of α‐SMA and COL1A1, along with inflammatory markers including IL‐1β and TNF‐α in the CNV‐C group (Figure S8B, Supporting Information). Taken together, these results demonstrate that CNV‐C can effectively mitigate liver fibrosis in vivo by combining its CCL2 adsorption capability with the loaded CUR.

### 2.6 CNV‐C Inhibits TGF‐β/Smad Signaling to Exert the Anti‐fibrotic Effect

**Figure 6:**
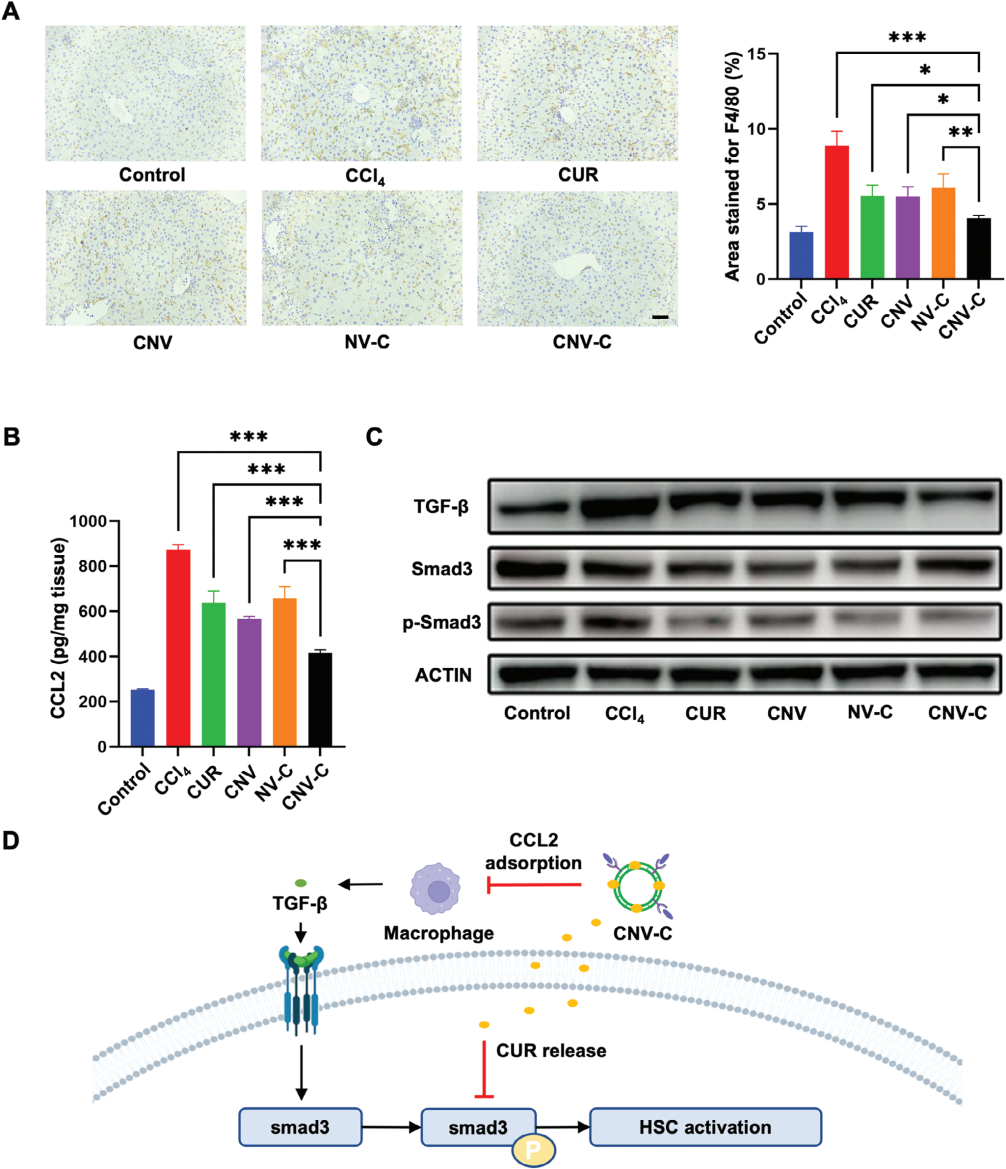
The therapeutic mechanism of CNV‐C in alleviating liver fibrosis. A) Representative images of F4/80 immunostaining in mice liver from indicated groups and the corresponding quantitative analysis (n = 5). Scale bars = 50 µm. B) CCL2 levels in the mice liver from indicated groups (n = 3). C) Western blot analysis of TGF‐β, Smad3, and p‐Smad3 expression in the mice liver from indicated groups. D) Schematic illustration of the mechanism underlying CNV‐C treatment in treating liver fibrosis. ^*^ *p* < 0.05; ^**^ *p* < 0.01; ^***^ *p* < 0.001.

To elucidate the therapeutic mechanism underlying CNV‐C treatment, macrophage infiltration in mouse livers was examined. Immunohistochemistry revealed a significant reduction in the infiltration of F4/80^+^ macrophages in the CNV‐C group compared to the other treatment groups (**Figure** **6**A). Additionally, the level of CCL2 in mouse livers was quantified using ELISA. As expected, the CNV‐C group exhibited the lowest CCL2 level among all treatment groups (Figure 6B). We noted that the macrophage infiltration and CCL2 levels were also decreased in the CUR group and NV‐C group, which may be attributed to the inhibition of CCL2 expression in Kupffer cells by CUR.^[^ ^26^ ^]^ These results suggest that CNV‐C diminishes macrophage infiltration by simultaneously adsorbing released CCL2 and suppressing its production. The reduced macrophage infiltration may subsequently lead to decreased release of fibrogenic cytokines, such as TGF‐β, which activates the TGF‐β signaling to promote HSC activation and ECM generation in liver fibrosis.^[^ ^2^ ^]^ Thus, we investigated whether CNV‐C treatment inhibits the TGF‐β/Smad pathway in fibrotic livers. As shown in Figure 6C, the level of TGF‐β in the CNV‐C group was significantly reduced. Moreover, phosphorylation of the downstream molecule Smad3 was also found to be inhibited after CNV‐C treatment. Although previous studies have reported the inhibitory effect of CUR on the TGF‐β/Smad signaling in liver fibrosis,^[^ ^17^ ^]^ we show here that this effect can be further enhanced by the removal of upstream fibrogenic mediators, thereby contributing to an improved therapeutic outcome (Figure 6D). Macrophages play a pivotal regulatory role in the progression of liver fibrosis.^[^ ^2^ ^]^ Beyond activating and proliferating HSCs through TGF‐β production, it has been reported that macrophages recruited in the liver can release other pro‐fibrotic factors such as TNF‐α, IL‐1β, and IL‐6, which also contribute to HSC activation.^[^ ^27^ ^]^ Therefore, CNV‐C treatment may also be involved in these downstream mechanisms. We are committed to unraveling these complex interactions in our future research endeavors. Furthermore, macrophage infiltration is commonly observed and contributes to the pathogenesis of other chronic diseases, such as atherosclerosis, pulmonary fibrosis, and even cancer.^[^ ^28^ ^]^ CNV‐C may exhibit therapeutical potential for these diseases, and further verification is needed to expand its clinical applicability.

## 3 Conclusion

In summary, we have developed a CUR‐loaded biomimetic nanodecoy, CNV‐C, through the genetic engineering of cellular nanovesicles. CNV‐C can absorb CCL2 specifically by over‐expressing CCR2 on its surface, which results in a significant inhibition of macrophage infiltration both in vitro and in vivo. This, in turn, leads to the reduced production of fibrogenic mediators. Moreover, CNV‐C improves the delivery of CUR to the fibrotic liver, where it effectively suppresses HSC activation by blocking the downstream TGF‐β/Smad signaling pathway. As a result, CNV‐C preserves the liver structure and mitigates collagen fiber proliferation in a CCl_4_‐induced liver fibrosis model. To our knowledge, this is the first study using genetically modified nanodecoy to treat liver fibrosis. Our findings may provide a new paradigm for the development of biomimetic nanomedicine against fibrosis and other inflammation‐related diseases.

## 4 Experimental Section

### Statistical Analysis

All data were statistically analyzed using GraphPad Prism 8 and are presented as mean ± SEM. The sample size (n) for each statistical analysis is indicated in the respective figure legend. An unpaired two‐tailed Student's t‐test was employed for comparisons between two groups, while a one‐way analysis of variance (ANOVA) was used for comparisons involving three or more groups. Asterisks denoted the range of *p*‐values: ^*^ for *p* < 0.05, ^**^ for *p* < 0.01, and ^***^ for *p* < 0.001. “n.s.” indicates no significant difference.

For detailed methods on materials, preparation, and characterization of CNV‐C, cell, and animal experiments, please refer to the Supporting Information.

## Conflict of Interest

The authors declare no conflict of interest.

## Supporting information

Supporting Information

## Data Availability

The data that support the findings of this study are available from the corresponding author upon reasonable request.
