# Supplementary material for: A Genetically Engineered Biomimetic Nanodecoy for the Treatment of Liver Fibrosis
Source: Adv Sci (Weinh). 2024 Aug 29;11(40):2405026. doi: 10.1002/advs.202405026 (PMC11516072; doi:10.1002/advs.202405026)
Supplement: Supplementary file 1 — Supporting Information [file ADVS-11-2405026-s001.docx]

Supporting Information

A Genetically Engineered Biomimetic Nanodecoy for the Treatment of Liver Fibrosis

Yang Du, Hao Ding, Yining Chen, Bingqiang Gao, Zhengwei Mao*, Weilin Wang*, Yuan Ding*

Y. Du^[+]^, H. Ding^[+]^, Y. Chen^[+]^, B. Gao, Z. Mao, W. Wang, Y. Ding

Department of Hepatobiliary and Pancreatic Surgery, the Second Affiliated Hospital, Zhejiang University School of Medicine, Hangzhou 310009, China

E-mail: dingyuan@zju.edu.cn (Y. Ding); wam@zju.edu.cn (W. Wang); zwmao@zju.edu.cn (Z. Mao)

Y. Du, H. Ding, Y. Chen, B. Gao, Z. Mao, W. Wang, Y. Ding

Key Laboratory of Precision Diagnosis and Treatment for Hepatobiliary and Pancreatic Tumor of Zhejiang Province, Hangzhou 310009, China

Y. Du, H. Ding, Y. Chen, B. Gao, W. Wang, Y. Ding

Research Center of Diagnosis and Treatment Technology for Hepatocellular Carcinoma of Zhejiang Province, Hangzhou 310009, China

Y. Du, H. Ding, Y. Chen, B. Gao, W. Wang, Y. Ding

National Innovation Center for Fundamental Research on Cancer Medicine, Hangzhou 310009, China

Y. Du, H. Ding, Y. Chen, B. Gao, W. Wang, Y. Ding

Cancer Center, Zhejiang University, Hangzhou 310058, China

Y. Du, H. Ding, Y. Chen, B. Gao, W. Wang, Y. Ding

ZJU-Pujian Research ＆ Development Center of Medical Artificial Intelligence for Hepatobiliary and Pancreatic Disease, Hangzhou 310058, China

Z. Mao

MOE Key Laboratory of Macromolecular Synthesis and Functionalization, Department of Polymer Science and Engineering, Zhejiang University, Hangzhou 310027, China

Z. Mao

State Key Laboratory of Transvascular Implantation Devices, Hangzhou 310009, China.

[+] These authors contributed equally to this work.

**Experimental Methods**

*Chemicals and Materials*

DMEM culture media and Penicillin-Streptomycin (100×) were purchased from Thermo Fisher (USA). Fetal bovine serum (FBS) was purchased from Biochannel (China). The PGMLV-CMV-Mouse_CCR2-mScarlet-PGK-Puro vector plasmid and puromycin were purchased from Genomeditech (China). The mouse CCR2 antibody was purchased from Affinity (USA). The mScarlet antibody was purchased from ChromoTek (German). The α-SMA antibody and COL1A1 antibody were purchased from Abcam (UK). The Na^+^/K^+^-ATPase antibody was purchased from ABclonal (USA). The actin antibody, HRP-conjugated Affinipure Goat Anti-Mouse IgG, and HRP-conjugated Affinipure Goat Anti-Rabbit IgG were purchased from Proteintech (USA). Tween-80, phosphate-buffered saline (PBS), and the ECL chemiluminescence kit were purchased from Servicebio (China). Bovine serum albumin (BSA), electrophoresis transfer buffer, dimethyl sulfoxide (DMSO), and Sirius red staining solution were purchased from Solarbio (China). Polybrene, membrane protein and cytoplasmic protein extraction kit, Phenylmethanesulfonyl fluoride (PMSF), Tris-HCl, DAPI, Hoechst 33342, 3,3-dioctadecyloxacarbocyanine perchlorate (DiO), 1,1'-dioctadecyl-3,3,3',3'-tetramethylindocarbocyanine perchlorate (DiI), BCA protein assay kit, 4 % paraformaldehyde fixative, QuickBlock™ Western blocking solution, crystal violet dyeing solution, regenerated cellulose dialysis bag (34 mm, 3.5 kDa), and Hematoxylin & eosin (H&E) staining kit were purchased from Beyotime (China). IR-780 iodine was purchased from J&K Scientific (China). The mouse MCP-1/CCL2 ELISA kit was purchased from CUSABIO (USA).

*Cell culture*

The human embryonic kidney 293T cell line (HEK 293T), mouse macrophage cell line (RAW264.7), human umbilical vein endothelial cells (HUVECs), and human hepatic stellate cell line (LX-2) were obtained from the National Collection of Authenticated Cell Cultures (Shanghai, China) and cultured according to ATCC guidelines. Cells were incubated in a humidified chamber with 5 % CO_2_ at 37 ℃. DMEM medium containing 10 % FBS and 1 % penicillin-streptomycin was used for cell culture.

*Construction and characterization of the CCR2 overexpressed cell line*

The gene for murine CCR2 was cloned into the PGMLV-CMV-Mouse_CCR2-mScarlet-PGK-Puro lentiviral vector plasmid. HEK 293T cells were transfected with the viral packaging vectors psPAX2 and PMD2G plasmid, along with the lentiviral vector plasmid, using Lipofectamine™ 2000 Transfection Reagent for 72 h. The lentivirus carrying murine CCR2 with a mScarlet tag in the supernatant was collected via ultracentrifuge (24000 g, 4 ℃, 4 h). Then, the HEK 293T cells were co-cultured with lentivirus and polybrene (10 μg/mL) for 48 h and underwent puromycin selection (2 µg/mL) to obtain the cell line stably expressing CCR2-mScarlet. To confirm the expression of CCR2-mScarlet in the engineered HEK 293T cell line, both pristine and engineered cells were seeded into µ-slide 4 wells at a density of 5 × 10^5^ and stained with Hoechst 33342 (10 min) and DiO (10 min). Subsequently, these cells were observed under a confocal laser scanning microscopy (CLSM, Zeiss, LSM 900, Germany).

*Generation and characterization of CNV*

CCR2-HEK293T cells (5 × 10^7^) were suspended in 1mL of Membrane Protein Extraction Reagent containing 1mM of PMSF in an ice bath for 15 min. The cell suspension underwent two freeze-thaw cycles and was then centrifuged at 1000 g for 10 min at 4 °C. The supernatant was collected and centrifuged at 16,000 g for 30 min at 4 °C to precipitate cell membrane fragments. The cell membranes were redispersed in PBS and quantified using a BCA kit to determine the protein content. Subsequently, the membranes were sequentially extruded through 400 nm and 200 nm polycarbonate membranes (GE Whatman, USA) using a mini-extruder (Avanti, USA) to obtain CNV. Western blot assays were performed to confirm the presence of CCR2 on CNV. In brief, CNV was lysed in RIPA buffer containing 1 % PMSF and heated to 70 °C for 10 min. Samples were then separated by SDS-PAGE and transferred onto nitrocellulose membranes. The membranes were blocked with a 5 % nonfat powdered milk solution for 30 min at room temperature and incubated with primary antibodies overnight at 4 °C. Finally, the membranes were incubated with secondary antibodies and imaged using an Amersham Imager 800 (GE Healthcare, USA).

*Preparation and characterization of CNV-C*

The prepared CNV was mixed with CUR in PBS at room temperature for 30 min with shaking. Unless otherwise specified, the mass ratio of CNV to CUR was set at 1:1. Then, the mixture was centrifuged at 16,000 g at 4°C to obtain CNV-C. TEM images were taken using a HITACHI HT7820 (Japan) at a voltage of 80 kV for morphology characterization. DLS and zeta potential analysis of CNV-C were conducted on a Zetasizer Lab (Malvern, UK). Western blot assays were performed to confirm the presence of CCR2 on CNV-C.

*Determination of drug loading capacity and encapsulation efficiency*

To measure the drug loading capacity of CNV-C, 0.2 mg of nanovesicles were resuspended in 2 mL of PBS and mixed with CUR at different concentrations (0.025, 0.05, 0.075, 0.1, and 0.125 mg/mL), shaking at room temperature for 30 min. The mixture was then centrifuged at 16,000 g for 30 min at 4 °C, and the CNV-C was resuspended in DMSO to measure the absorbance at 436 nm using a UV spectrophotometer. The concentration of CUR in the CNV-C was determined based on the CUR standard curve. The drug loading capacity was calculated as the percentage of CUR mass loaded relative to the total mass of the CNV-C, while encapsulation efficiency was calculated as the percentage of CUR mass loaded relative to the feeding mass of CUR. For the *in vitro* drug release assay, the release of CUR from CNV-C was measured using a dialysis method. 2.5 mg of CNV-C dispersed in 4 mL of PBS were transferred to a dialysis bag (MWCO: 3.5 kDa) and placed in 300 mL of PBS containing Tween-80 (1 % v/v) and ethanol (30 % v/v). This system was then incubated in a water bath at 37 °C with constant stirring. At time intervals of 1, 2, 3, 4, 5, 6, 12, 24, 36, 48, 60, and 72 h, 10 mL of medium was collected and replaced with the same volume of fresh medium. The absorbance of the medium was measured at 436 nm, and the concentrations of released CUR in the medium were calculated based on the CUR standard curve (Figure S1).

*In vitro CCL2 adsorption*

To investigate the capacity of CNV-C to adsorb CCL2, serum samples enriched with CCL2 were collected from the C57BL/6 mice 8 h after intraperitoneal injection of APAP (300 mg/kg). A 100 μL aliquot of CCL2-enriched serum was mixed with NV, CNV, or CNV-C at nanovesicle concentrations of 0.15, 0.3, and 0.6 mg/mL. These mixtures were incubated with shaking at 37 °C for 30 min and then centrifuged at 16,000 g for 30 min at 4 °C to remove the nanovesicles. The CCL2 concentrations in the supernatant of each group were subsequently quantified using a CCL2 ELISA kit.

*In vitro inhibition of macrophage migration*

2.0×10^4^ HUVECs were seeded into 24-well transwell filters and then incubated when cell confluency reached 100 %. RAW264.7 cells were resuspended in serum-free DMEM medium at a concentration of 2.5×10^5^ cells/mL. The cell suspension (200 μL/well) was added to the upper chamber. For the control group, 700 μL of complete medium containing 10 % FBS was added to each well in the lower chamber; For the Serum group, 700 μl of complete medium containing CCL2-enriched serum was added to each well in the lower chamber; For the Serum + NV, Serum + CNV, and Serum + CNV-C groups, 700 μl of complete medium containing CCL2-enriched serum after treatment with the indicated nanovesicles (0.3 mg/mL) was added to each well in the lower chamber. After 12 h of incubation, RAW264.7 cells were fixed with 4 % paraformaldehyde for 10 min and then washed twice with PBS for 2 min each time. Next, the cells were stained with crystal violet staining solution for 10 min and washed thoroughly with PBS. Non-migrated cells on the upper chamber side were wiped off using a cotton swab. Migrated cells on the lower chamber side were counted using a microscope.

*In vitro inhibition of HSC activation*

LX-2 cells were inoculated in a 6-well plate and divided into 6 groups: (1) Control group; (2) TGF-β group (10 ng/mL); (3) TGF-β + CUR group (11 μg/mL of CUR); (4) TGF-β + CNV group (13 μg/mL of CNV) ; (5) TGF-β + NV-C group (24 μg/mL of NV-C, equivalent to 11 μg/mL of CUR); (6) TGF-β + CNV-C group (24 μg/mL of CNV-C, equivalent to 11 μg/mL of CUR). After 24 h of incubation, the cells were collected to detect α-SMA expression by immunofluorescent staining. In addition, Western blot assays were used to measure the expression of α-SMA, COL1A1, Smad3, and p-Smad3 after the indicated treatments.

*In vivo pharmacokinetics and biodistribution*

All animal experiments were carried out following the guidelines set by the Institute of Laboratory Animal Resources. The study protocol received approval from the Ethics Committee of the Second Affiliated Hospital of Zhejiang University School of Medicine (2023-ND-D-34). C57BL/6 mice (n = 3) were administered a single intravenous injection of free CUR or CNV-C. Plasma samples were collected from the inner canthus veniplex at time intervals of 7.5, 15, 30, 60, 120, 180, 360, 720, and 1140 min post-injection and dissolved in DMSO. The fluorescence intensity of CUR was measured at λex/em = 430/530 nm. Serum CUR levels were calculated using the standard curve of CUR fluorescence intensity (y = 2500.9x + 158.0, R^2^ = 0.998). PK parameters were calculated using PKSOLVER. To observe the *in vivo* biodistribution of CNV-C, CUR was replaced with the near-infrared fluorescent dye IR780 in the CNV. C57BL/6 mice were randomly divided into two groups: free IR780 and CNV-IR780 groups (n = 5) and were depilated 1 day in advance. Each group received an injection of free IR780 or CNV-IR780 via the tail vein at a dose of 0.5 mg/kg IR780. Whole-body fluorescence imaging of the mice was performed using an in vivo animal imaging system (PerkinElmer, USA), with the excitation/emission wavelength set to 745/808 nm. Fluorescent images were captured with isoflurane-anesthetized mice at different time points (2, 6, 12, and 24 h). For *ex vivo* tissue imaging, the mice were euthanized 24 h post-injection, and the major organs (heart, lungs, liver, kidneys, and spleen) were collected for imaging. Quantification and analysis were carried out using Living Imaging software.

*In vivo cellular uptake and elimination*

To investigate the elimination of CNV-C, CNV-IR780 was administrated to C57BL/6 mice (n = 4). Major organs (heart, liver, spleen, lung, and kidney) were harvested at indicated time points (24, 36, 48, and 72 h post-injection) for in vivo fluorescence imaging. The fluorescence intensity was quantified using Living Imaging software. To investigate the cellular uptake of CNV-C in the liver, Cy5.5-labeled CNV-C was administrated to C57BL/6 mice. Livers were harvested at 24 and 72 h post-injection for immunofluorescence staining. Liver sections were stained with ALB, F4/80, or α-SMA antibodies at 4 ℃ overnight. Subsequently, the Goat Anti-Rabbit IgG H&L (Alexa Fluor® 488) antibody was sequentially added and incubated at room temperature for 1 h. DAPI was applied to stain the nuclei. The slices were then observed under fluorescence microscopy.

*In vivo anti-fibrosis therapy*

C57BL/6 mice were randomly assigned to six different groups, each comprising 5 mice: the Control group, CCl_4_ model group, CUR group (8 mg/kg), CNV group (10 mg/kg), NV-C group (18 mg/kg, equivalent CUR content 8 mg/kg), and CNV-C group (18 mg/kg, equivalent CUR content 8 mg/kg). For the CUR group, CUR was dissolved in PBS containing 10 % DMSO and 10 % SBE-β-CD, while the drug formulations in the other groups were prepared in PBS. Except for the control group, mice in the remaining five groups were intraperitoneally injected with 1 mL/kg CCl_4_ (1:4 diluted in olive oil) twice a week for 6 consecutive weeks to induce liver fibrosis. The control group received an equal volume of PBS through intraperitoneal injection. Starting from the 4th week, the CUR group, CNV group, NV-C group, and CNV-C group were intravenously injected with corresponding doses of drug formulations twice a week, while the control group and CCl_4_ model group received an equal volume of PBS solution. At the end of the 6th week, the mice were anesthetized with isoflurane, and their liver tissues and serum samples were collected. Histopathological analyses using H&E staining, Sirius Red staining, and immunohistochemical staining for α-SMA and COL1A1 were employed to assess the degree of liver fibrosis. F4/80 immunostaining was used to evaluate macrophage infiltration. Western blot assays were conducted to measure the expression of α-SMA, COL1A1, TGF-β, Smad3, p-Smad3, and Actin in the liver. Changes in mRNA expression of α-SMA, COL1A1, IL-1β, and TNF-α were determined by qPCR. The levels of CCL2 in the liver were detected using a CCL2 ELISA kit.

*Biosafety evaluation*

C57BL/6 mice were randomly divided into two groups. For short-term biosafety evaluation (n = 4), one group received PBS, while the other received CNV-C via a single tail vein injection. For long-term biosafety evaluation (n = 5), one group received PBS, while the other received CNV-C via tail vein injection twice a week for 3 weeks. The injection dose for CNV-C was 18 mg/kg, with an equivalent CUR content of 8 mg/kg. After 1 day or 3 weeks of injections, the mice were euthanized, and their hearts, lungs, livers, spleens, and kidneys were harvested for H&E staining analysis. Additionally, blood samples were collected for biochemical analysis.

*Statistical analysis*

All data were statistically analyzed using GraphPad Prism 8 and are presented as mean ± SEM. The sample size (n) for each statistical analysis is indicated in the respective figure legend. An unpaired two-tailed Student's t-test was employed for comparisons between two groups, while a one-way analysis of variance (ANOVA) was used for comparisons involving three or more groups. Asterisks denoted the range of p-values: * for p < 0.05, ** for p < 0.01, and *** for p < 0.001. “n.s.” indicates no significant diﬀerence.

**Supporting Figures**

Figure S1. (A) The standard curve of CUR. (B) Photograph of CUR in DMSO, and CNV and CNV-C in PBS. (C) The loading capacity of CUR in CNV-C at different feeding ratios (n = 3). (D) The encapsulation efficiency of CUR in CNV-C at different feeding ratios (n = 3).

Figure S2. In vitro stability of CNV-C. (A) Particle size of CNV-C in PBS and 10 % FBS-DMEM (n = 3). (B) Zeta potential of CNV-C in PBS and 10 % FBS-DMEM (n = 3).


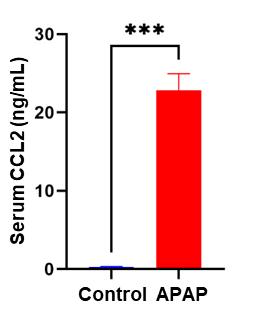


Figure S3. Serum CCL2 levels in mice at 8 h after 300 mg/kg APAP administration (n = 3). ***, p < 0.001.

Figure S4. Western blot analysis of Smad3 and p-Smad3 expression in LX-2 cells after different treatments

Figure S5. In vivo cellular uptake and elimination of CNV-C. (A) Representative ex vivo fluorescence images of major organs (heart, liver, spleen, lung, and kidney) harvested at the indicated time points after CNV-IR780 administration, with quantitative analysis of the fluorescence intensity in the major organs (n = 4). (B) Representative fluorescence microscope images of Cy5.5-labeled CNV-C in liver sections at the indicated time points. Cy5.5-labeled CNV-C (red), DAPI (blue), ALB, α-SMA, and F4/80 (green). Scale bar = 100 μm.

Figure S6. Short-term biosafety evaluation of CNV-C. (A) Serum biochemical indexes (serum ALT, AST, ALP, UREA, CRE-J, TBIL, PLT, HGB, RBC, Mon, Neu, and WBC levels) of mice after CNC-C treatment (n = 4). (B) Representative images of H&E-stained organ slices from indicated groups. Scale bars = 100 μm. n.s., not significant (p > 0.05).

Figure S7. Long-term biosafety evaluation of CNV-C. (A) Serum biochemical indexes (serum ALT, AST, ALP, UREA, CRE-J, TBIL, PLT, HGB, RBC, Mon, Neu, and WBC levels) of mice after CNC-C treatment (n = 5). (B) Representative images of H&E-stained organ slices from indicated groups. Scale bars = 100 μm. n.s., not significant (p > 0.05).

Figure S8. (A) Western blot analysis of α-SMA and COL1A1 expression in the mice liver from indicated groups. Relative gene expression of α-SMA (B), COL1A1 (C), IL-1β (D), and TNF-α (E) in mice liver from indicated groups (n = 3). *, p < 0.05; **, p < 0.01; ***, p < 0.001.
